# Succinylated Wheat Germ Agglutinin Colocalizes with the Toxoplasma gondii Cyst Wall Glycoprotein CST1

**DOI:** 10.1128/mSphere.00031-20

**Published:** 2020-03-04

**Authors:** Rebekah B. Guevara, Barbara A. Fox, David J. Bzik

**Affiliations:** a Department of Microbiology and Immunology, The Geisel School of Medicine at Dartmouth, Lebanon, New Hampshire, USA; University of Georgia

**Keywords:** *Toxoplasma gondii*, cysts, chronic infection, cyst wall, cyst matrix, bradyzoite differentiation, cyst development, *N*-acetylglucosamine, s-WGA

## Abstract

The glycosylated mucin domain of the Toxoplasma gondii cyst wall glycoprotein CST1 is heavily stained by Dolichos biflorus agglutinin, a lectin that binds to *N*-acetylgalactosamine. The cyst wall is also heavily stained by the chitin binding lectin succinylated wheat germ agglutinin (s-WGA), which selectively binds to *N-*acetylglucosamine-decorated structures. Here, we tracked the localization of *N-*acetylglucosamine-decorated structures that bind to s-WGA in immature and mature *in vitro* cysts. s-WGA localization was observed at the cyst periphery 6 h after the differentiation of the tachyzoite-stage parasitophorous vacuole. By day 1 and at all later times after differentiation, s-WGA was localized in a continuous staining pattern at the cyst wall. Coinciding with the maturation of the cyst matrix by day 3 of cyst development, s-WGA also localized in a continuous matrix pattern inside the cyst. s-WGA localized in both the outer and inner layer regions of the cyst wall and in a continuous matrix pattern inside mature 7- and 10-day-old cysts. In addition, s-WGA colocalized in the cyst wall with CST1, suggesting that *N*-acetylglucosamine- and *N*-acetylgalactosamine-decorated molecules colocalized in the cyst wall. In contrast to CST1, GRA4, and GRA6, the relative accumulation of the molecules that bind s-WGA in the cyst wall was not dependent on the expression of GRA2. Our results suggest that GRA2-dependent and GRA2-independent mechanisms regulate the trafficking and accumulation of glycosylated molecules that colocalize in the cyst wall.

**IMPORTANCE** Chronic Toxoplasma gondii infection is maintained in the central nervous system by thick-walled cysts. If host immunity wanes, cysts recrudesce and cause severe and often lethal toxoplasmic encephalitis. Currently, there are no therapies to eliminate cysts, and little biological information is available regarding cyst structure(s). Here, we investigated cyst wall molecules recognized by succinylated wheat germ agglutinin (s-WGA), a lectin that specifically binds to *N-*acetylglucosamine-decorated structures. *N-*Acetylglucosamine regulates cell signaling and plays structural roles at the cell surface in many organisms. The cyst wall and cyst matrix were heavily stained by s-WGA in mature cysts and were differentially stained during cyst development. The relative accumulation of molecules that bind to s-WGA in the cyst wall was not dependent on the expression of GRA2. Our findings suggest that glycosylated cyst wall molecules gain access to the cyst wall via GRA2-dependent and GRA2-independent mechanisms and colocalize in the cyst wall.

## Abstract (precis)

Chronic Toxoplasma gondii infection is maintained in the central nervous system by thick-walled cysts. If host immunity wanes, cysts recrudesce and cause severe and often lethal toxoplasmic encephalitis. Currently, there are no therapies to eliminate cysts, and little biological information is available regarding cyst structure(s). Here, we investigated cyst wall molecules recognized by succinylated wheat germ agglutinin (s-WGA), a lectin that specifically binds to *N-*acetylglucosamine-decorated structures. *N-*Acetylglucosamine regulates cell signaling and plays structural roles at the cell surface in many organisms. The cyst wall and cyst matrix were heavily stained by s-WGA in mature cysts and were differentially stained during cyst development. The relative accumulation of molecules that bind to s-WGA in the cyst wall was not dependent on the expression of GRA2. Our findings suggest that glycosylated cyst wall molecules gain access to the cyst wall via GRA2-dependent and GRA2-independent mechanisms and colocalize in the cyst wall.

## INTRODUCTION

Toxoplasma gondii is a protozoan pathogen that chronically infects one-third of the global human population (1). Lifelong infection is established by the formation of thick-walled tissue cysts, which mediate parasite transmission (2). Humans are infected by the ingestion of tissue cysts in undercooked meat or oocysts in water or unwashed food (3, 4). *Toxoplasma* infection causes severe ocular infections of the eye (5), and primary infection during pregnancy causes severe congenital defects in the newborn (6, 7). AIDS, cancer, and transplant patients with weakened immunity are susceptible to reactivated chronic infection that causes life-threatening toxoplasmic encephalitis (8, 9). Therapies with the ability to target the cyst stage are not yet available.

During acute infection, tachyzoite-stage parasites replicate within a transient parasitophorous vacuole (PV) that is breached when tachyzoites egress to invade new host cells (10, 11). In chronic infection, bradyzoite-stage parasites reside in a more permanent structure, termed the cyst. While the biology of cyst formation is not yet well understood, prominent cyst structures include the limiting cyst membrane, the cyst wall, and the cyst matrix, which surround the bradyzoite-stage parasites. After tachyzoite-to-bradyzoite-stage differentiation is triggered, the PV membrane (PVM) develops into the cyst membrane (12). A 200- to 850-nm-thick cyst wall forms beneath the cyst membrane (13, 14). Within 6 h after differentiation, cyst wall cargo is already accumulating at the cyst periphery (12, 15). In mature cysts, the cyst wall is organized into two distinct filamentous layers, a more densely compacted outer layer beneath a limiting cyst membrane and a less densely compacted inner layer that faces the cyst matrix (13). The major cyst wall glycoprotein CST1, at least 22 dense granule (GRA) proteins, and other proteins, including CST4, BPK1, MAG1, MCP3, MCP4, and MYR1, were identified in the cyst wall/membrane of *in vitro* cysts (16). In addition, CST1, GRA2, GRA5, GRA6, GRA7, and GRA12 occupy both cyst wall layers, while GRA1, GRA4, and GRA9 occupy only the inner layer of the cyst wall in mature *in vitro* cysts (12, 15). Genetic deletion of cyst membrane- and cyst wall-associated GRA proteins significantly reduced cyst burdens in mice infected with *Toxoplasma* (17–19), suggesting that cyst membrane- and cyst wall-associated GRA proteins were important for the formation and durability of cysts.

The cyst matrix is an intricate environment decorated with soluble components, filamentous materials, vesicles, and membranous tubules (13). The cyst matrix membranous tubules, which also extend into the cyst wall, were previously suggested to be structures that are similar to the intravacuolar network (IVN) membranes characterized in the tachyzoite PV and were therefore named the intracyst network (ICN) (13, 14). A complex of IVN-associated GRAs, GRA2/GRA4/GRA6, interacts with the membranous tubules (20, 21). IVN-associated GRA2 induces the formation of curved tubules from vesicular material secreted from the posterior end of the parasite, and GRA6 stabilizes the curvature of the newly formed membranous tubules (21). The cyst matrix forms 3 days after differentiation and fills the spaces between the bradyzoites and the cyst wall (15). In mature *in vitro* cysts, GRA1, GRA4, GRA6, and GRA9 established a continuous matrix pattern, while GRA2 and GRA12 were isolated in distinct puncta in the cyst matrix (15). GRA2 regulates the development of the cyst wall and the organization of the cyst matrix (15).

Cysts isolated from chronically infected mice were previously assessed with a variety of lectins to identify carbohydrate structures associated with the cyst wall (22–24). The cyst wall is intensely stained by two lectins, Dolichos biflorus agglutinin (DBA) and succinylated wheat germ agglutinin (s-WGA) (23, 24). DBA selectively recognizes terminal α-linked *N*-acetylgalactosamine structures (25). DBA preferentially binds to the mucin domain of CST1, the major cyst wall glycoprotein (19). The CST1-mucin domain is *O*-linked *N*-acetylgalactosamine glycosylated by ppGalNAc-T2 and -T3 (26). Deletion of the *Toxoplasma* nucleotide sugar transporter (TgNST1) ablated cyst wall recognition by DBA as well as s-WGA (27), suggesting that *Toxoplasma* steals nucleotide sugars from the host cell. A second *O*-linked glycosylated mucin domain-containing cyst wall glycoprotein was recently identified as SRS13 (28). However, SRS13 is a low-abundance cyst wall protein (16), which is consistent with the observation that DBA staining of the cyst wall was abolished in cysts that lacked the expression of CST1 (19). Genetic deletion of CST1 reduced the number of brain cysts, and these CST1-deficient cysts exhibited a fragile-cyst phenotype characterized by a thin cyst wall and cysts that were easily disrupted by mechanical force (19).

In contrast to the *N*-acetylgalactosamine specificity of DBA, the chitin binding lectin s-WGA selectively binds to *N-*acetylglucosamine-modified structures (29). Lectin overlay experiments previously revealed that s-WGA selectively recognized an ∼48-kDa cyst wall-localized glycoprotein that is not related to CST1 (23). Treatment of *Toxoplasma* cysts with chitinase, an enzyme specific for a β-1,4-linked polymer of *N-*acetylglucosamine, degraded the cyst wall and released bradyzoites (24, 30). In addition, s-WGA failed to stain the cyst wall of cysts that were pretreated with chitinase (24). Together, these observations suggested that s-WGA binds to an *N-*acetylglucosamine-modified glycoprotein that is essential for the stability of the cyst wall.

Exposure of tachyzoite PVs to low CO_2_ and high pH triggers tachyzoite-to-bradyzoite differentiation (31, 32), and this *in vitro* differentiation method can produce mature, orally infectious cysts (33) that possess the characteristic cyst structures of *in vivo* cysts isolated from brains of infected mice (34). Here, we investigated s-WGA staining of cysts to (i) localize the *N-*acetylglucosamine-modified molecules that bind s-WGA during cyst development and (ii) test whether the accumulation of the s-WGA-binding *N-*acetylglucosamine-modified molecules in the cyst wall is regulated by GRA2. Our results show that s-WGA recognized *N-*acetylglucosamine-modified molecules at the cyst periphery/wall throughout cyst development and in the cyst matrix by day 3 after differentiation. The deletion of GRA2 did not affect the trafficking or cyst wall accumulation of the molecules that bind s-WGA in the cyst wall, and the cyst wall molecule(s) that binds s-WGA was colocalized with GRA4, GRA6, and CST1.

## RESULTS

### s-WGA localizes to the cyst wall and cyst matrix during cyst development.

**FIG 1:**
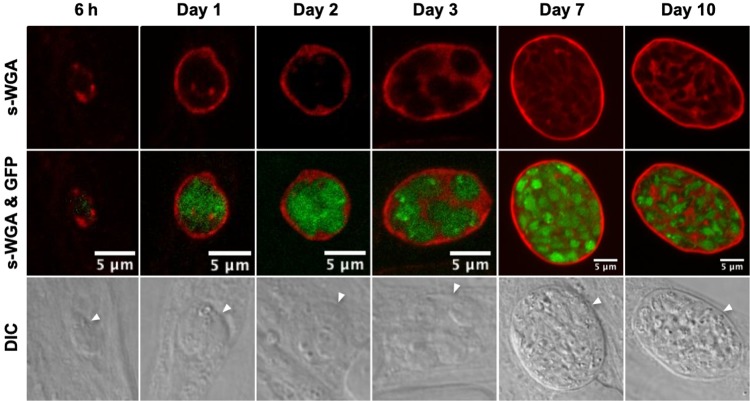
s-WGA lectin stains the cyst periphery, cyst wall, and cyst matrix throughout cyst development. *In vitro* cysts derived from the parental PruΔ*ku80* strain were differentiated for 6 h, 1 day, 2 days, 3 days, 7 days, or 10 days. Cysts were located using differential interference contrast (DIC) microscopy and imaged by confocal microscopy. The presence of bradyzoites inside cysts was verified by locating parasite nuclei with 4′,6-diamidino-2-phenylindole (DAPI) staining (not shown) and verifying that each parasite nucleus was surrounded by the expression of cytosolic GFP (GFP^+^ bradyzoites). Cysts were stained with biotinylated s-WGA. Panels show s-WGA, s-WGA and GFP, and DIC (cyst walls are indicated by white arrowheads). The number of cysts analyzed was 8 to 20. Representative images are shown.

The lectin s-WGA selectively recognizes an unidentified ∼48-kDa cyst wall glycoprotein (23). We used a streptavidin-biotin labeling scheme to track s-WGA localization during cyst development. Since streptavidin was previously observed to label endogenously biotinylated proteins in the apicoplast of tachyzoite-stage parasites (35–37), we performed control experiments to determine whether bradyzoite apicoplast biotin was detected. While the bradyzoite apicoplast was detected in cysts that were labeled only with streptavidin (see Fig. S1A in the supplemental material), the bradyzoite apicoplast was not detected in cysts that were initially stained with biotinylated s-WGA and then subsequently stained with streptavidin (Fig. S1B). Using this modified labeling scheme, we tracked the locations of the *N-*acetylglucosamine-modified molecules that bind to s-WGA in 6-h-, 1-day-, 2-day-, 3-day-, 7-day-, and 10-day-old *in vitro* cysts. Green fluorescent protein-positive (GFP^+^) bradyzoites were visible within developing cysts (Fig. 1). s-WGA staining was observed by as early as 6 h and at all later times of cyst development. The s-WGA staining patterns indicated that (i) at 6 h, s-WGA staining was observed as puncta at the cyst periphery; (ii) at 1 and 2 days, s-WGA was continuous around the cyst periphery; (iii) at 3 days, s-WGA was at the cyst periphery and visibly stained the cyst matrix; and (iv) at 7 and 10 days, s-WGA was at the cyst wall and established a continuous matrix pattern in the cyst (Fig. 1). In 7- and 10-day-old mature cysts, the cyst wall was visualized as a distinct structure by differential interference contrast (DIC) microscopy (Fig. 1). These observations suggested that the *N-*acetylglucosamine-modified molecules recognized by s-WGA were expressed early in the developing cyst and were associated with the cyst periphery/wall and cyst matrix during cyst development and cyst maturation.

10.1128/mSphere.00031-20.1 FIG S1 Streptavidin labels biotinylated proteins inside the developing cyst, and PruΔ*gra2* lacks expression of GRA2. *In vitro* cysts derived from the parental PruΔ*ku80* (A) or Δ*gra2* (B) strain were differentiated for 1 day or 2 days. Cysts were located using differential interference contrast (DIC) microscopy and imaged by confocal microscopy. The presence of bradyzoites inside cysts was verified by locating parasite nuclei with 4′,6-diamidino-2-phenylindole (DAPI) staining and verifying that each parasite nucleus was surrounded by the expression of cytosolic GFP (GFP^+^ bradyzoites). DAPI stains parasite and host nuclei. Cysts were stained with streptavidin and goat anti-mouse IgG antibody (A) or biotinylated s-WGA and anti-GRA2 antibody (B) (*n *= 9 and *n *= 12 [A] and *n *= 5 and *n *= 16 [B] for 1 day and 2 days, respectively). Bars = 5 μm. Download FIG S1, TIF file, 1.7 MB. Copyright © 2020 Guevara et al. 2020 Guevara et al. This content is distributed under the terms of the Creative Commons Attribution 4.0 International license.

### The localization of s-WGA in immature cysts is not dependent on GRA2 expression.

**FIG 2:**
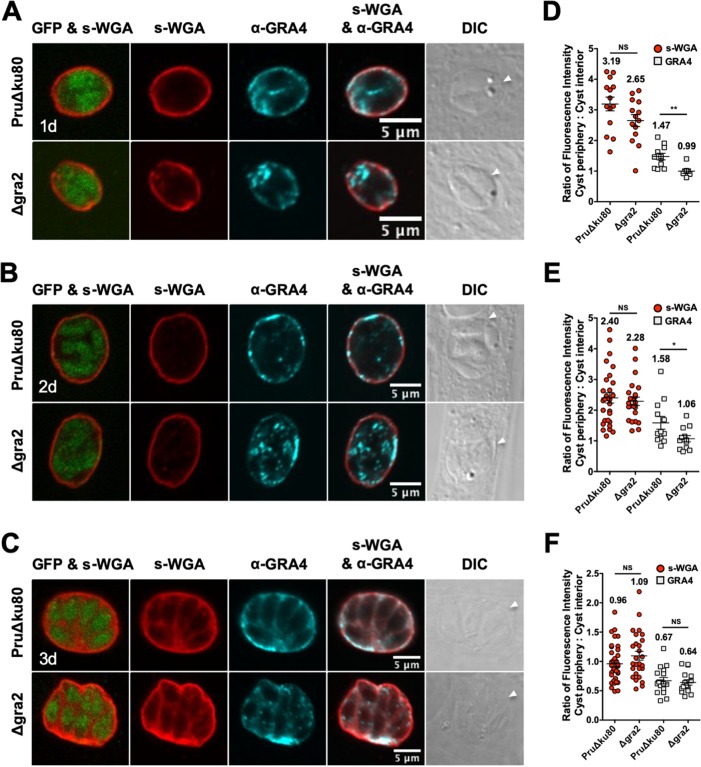
s-WGA colocalizes with GRA4 at the cyst periphery, and s-WGA accumulation at the cyst periphery is GRA2 independent in immature cysts. PruΔ*ku80*- and Δ*gra2*-infected human foreskin fibroblast (HFF) cells on coverslips were treated under bradyzoite-inducing conditions to differentiate *in vitro* cysts for 1 day, 2 days, or 3 days. Cysts were located using DIC microscopy and imaged by confocal microscopy. (A to C) The presence of bradyzoites inside cysts was verified by locating parasite nuclei with DAPI staining (not shown) and verifying that each parasite nucleus was surrounded by the expression of cytosolic GFP (GFP^+^ bradyzoites). Cysts were stained with biotinylated s-WGA and anti-GRA4 antibody. Panels show GFP and s-WGA, s-WGA, anti-GRA4, s-WGA and anti-GRA4, and DIC (cyst walls are indicated by white arrowheads). s-WGA colocalized with GRA4 in 100% of cysts evaluated at day 1 (*n *= 11) (A), day 2 (*n *= 12) (B), and day 3 (*n *= 17) (C). (D to F) Fluorescence intensities of s-WGA and GRA4 were measured at the cyst periphery and within the cyst (cyst interior) in PruΔ*ku80* and Δ*gra2* cysts at day 1 (D), day 2 (E), and day 3 (F). Data are plotted as the ratio of the mean fluorescence intensity at the cyst periphery to that within the cyst interior ± SEM. The numbers of cysts analyzed for the parental PruΔ*ku80* and Δ*gra2* strains for s-WGA and GRA4 are as follows: s-WGA (*n* = 14, *n* = 14 cysts) and GRA4 (*n* = 11, *n* = 7 cysts) at day 1 (D), s-WGA (*n* = 29, *n* = 24 cysts) and GRA4 (*n* = 12, *n* = 12 cysts) at day 2 (E), and s-WGA (*n* = 36, *n* = 27 cysts) and GRA4 (*n* = 17, *n* = 16 cysts) at day 3 (F). The numerical ratio for the mean fluorescence intensity is labeled for s-WGA and GRA4. *P* values were calculated using Student’s *t* test (*, *P < *0.05; **, *P < *0.01; NS, not significant).

**FIG 3:**
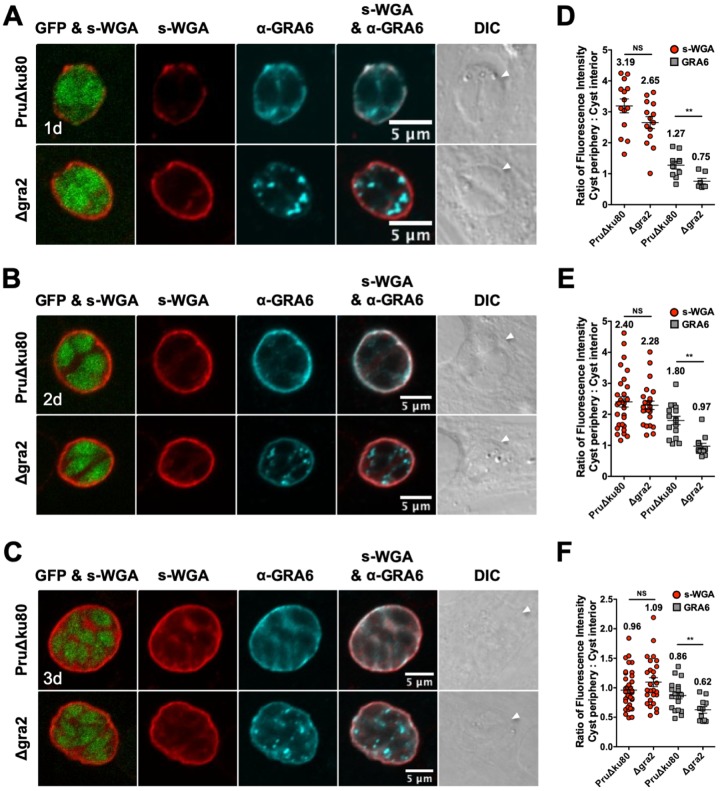
s-WGA colocalizes with GRA6 at the cyst periphery, and s-WGA accumulation at the cyst periphery is GRA2 independent in immature cysts. PruΔ*ku80*- and Δ*gra2*-infected HFF cells on coverslips were treated under bradyzoite-inducing conditions to differentiate *in vitro* cysts for 1 day, 2 days, or 3 days. Cysts were located using DIC microscopy and imaged by confocal microscopy. (A to C) The presence of bradyzoites inside cysts was verified by locating parasite nuclei with DAPI staining (not shown) and verifying that each parasite nucleus was surrounded by the expression of cytosolic GFP (GFP^+^ bradyzoites). Cysts were stained with biotinylated s-WGA and anti-GRA6 antibody. Panels show GFP and s-WGA, s-WGA, anti-GRA6, s-WGA and anti-GRA6, and DIC (cyst walls are indicated by white arrowheads). s-WGA was colocalized with GRA6 in 100% of cysts evaluated at day 1 (*n *= 10) (A), day 2 (*n *= 16) (B), and day 3 (*n *= 19) (C). (D to F) Fluorescence intensities of s-WGA and GRA6 were measured at the cyst periphery and within the cyst (cyst interior) in PruΔ*ku80* and Δ*gra2* cysts at day 1 (D), day 2 (E), and day 3 (F). Data are plotted as the ratio of the mean fluorescence intensity at the cyst periphery to that within the cyst interior ± SEM. The numbers of cysts analyzed for the parental PruΔ*ku80* and Δ*gra2* strains for s-WGA and GRA6 are as follows: s-WGA (*n* = 14, *n* = 14 cysts) and GRA6 (*n* = 10, *n* = 7 cysts) at day 1 (D), s-WGA (*n* = 29, *n* = 24 cysts) and GRA6 (*n* = 16, *n* = 12 cysts) at day 2 (E), and s-WGA (*n* = 36, *n* = 27 cysts) and GRA6 (*n* = 19, *n* = 11 cysts) at day 3 (F). The numerical ratio for the mean fluorescence intensity is labeled for s-WGA and GRA6. *P* values were calculated using Student’s *t* test (**, *P < *0.01; NS, not significant).

The relative accumulation of GRA4, GRA6, and CST1 at the cyst periphery/wall was delayed in Δ*gra2* cysts (15). To measure whether the relative accumulation of the *N-*acetylglucosamine-modified molecules that bind to s-WGA at the cyst periphery/wall was also regulated by GRA2, immature cysts were differentiated for 1 day, 2 days, or 3 days, and s-WGA colocalization with GRA4 (Fig. 2) or GRA6 (Fig. 3) was assessed in parental PruΔ*ku80* and Δ*gra2* cysts that lacked the expression of GRA2 (Fig. S1B). GFP^+^ bradyzoites were visible in s-WGA-stained cysts. In 1- and 2-day-old PruΔ*ku80* cysts, s-WGA preferentially localized at the cyst periphery and colocalized with GRA4 (Fig. 2A and B) and GRA6 (Fig. 3A and B). In contrast, in 1- and 2-day-old Δ*gra2* cysts, s-WGA weakly colocalized at the cyst periphery with GRA4 and GRA6. In 3-day-old PruΔ*ku80* cysts, s-WGA colocalized at the cyst periphery and also in the cyst matrix with GRA4 (Fig. 2C) and GRA6 (Fig. 3C), and this colocalization was reduced in Δ*gra2* cysts. In 3-day-old PruΔ*ku80* cysts, GRA4 (Fig. 2C) and GRA6 (Fig. 3C) also exhibited a continuous matrix staining pattern that transitioned to a less continuous punctum-speckled matrix pattern in Δ*gra2* cysts. In contrast, s-WGA displayed a continuous matrix pattern in both PruΔ*ku80* and Δ*gra2* cysts.

To quantify the location of s-WGA, GRA4, and GRA6 in immature cysts, we measured the fluorescence intensity at the cyst periphery relative to that at the cyst interior using a Fiji macro (15). While the cyst periphery/cyst interior ratios of GRA4 (Fig. 2D to F) and GRA6 (Fig. 3D to F) were significantly reduced in Δ*gra2* cysts compared to parental PruΔ*ku80* cysts, the lack of GRA2 expression did not affect the s-WGA cyst periphery/cyst interior ratio. In addition, the cyst periphery/cyst interior fluorescence intensity ratios of GRA4 (Fig. 2D and E), GRA6 (Fig. 3D and E), and s-WGA (Fig. 2D and E and Fig. 3D and E) were >1.0 in 1- and 2-day-old cysts, indicating that these molecules were preferentially localized in the cyst wall early in cyst development (15). Compared to 1- and 2-day-old cysts, the cyst periphery/cyst interior fluorescence intensity ratios of GRA4 (Fig. 2F), GRA6 (Fig. 3F), and s-WGA (Fig. 2F and Fig. 3F) were decreased in 3-day-old cysts, indicating that these molecules were also prominent in the cyst matrix by day 3 of cyst development.

To quantitatively assess the location(s) of GRA4, GRA6, and s-WGA, we measured the cyst fluorescence intensity profiles of GRA4 and GRA6 relative to s-WGA using the Fiji macro (15). The fluorescence intensity peaks of GRA4 (Fig. S2A to C) and GRA6 (Fig. S2D to F) overlapped that of s-WGA, indicating their presence at the cyst periphery in PruΔ*ku80* cysts. In contrast, in Δ*gra2* cysts, the fluorescence intensity peaks of GRA4 and GRA6 were shifted to the right (cyst matrix) of the s-WGA peak, confirming a reduced localization of GRA4 (Fig. S2A to C) and GRA6 (Fig. S2D to F) in the Δ*gra2* cyst wall (15). However, the fluorescence intensity of s-WGA at the cyst periphery/wall was not affected in 1-, 2-, or 3-day-old Δ*gra2* cysts.

10.1128/mSphere.00031-20.2 FIG S2 s-WGA accumulation at the cyst periphery is GRA2 independent in immature cysts. Fluorescence intensity profiles of representative cysts, shown in Fig. 2A to C for GRA4 and in Fig. 3A to C for GRA6, were generated to quantify the location of s-WGA relative to GRA4 (A to C) or GRA6 (D to F) at the cyst wall at day 1, day 2, and day 3 in parental PruΔ*ku80* and Δ*gra2* strains. Dotted black lines define the cyst wall region. The dotted red line indicates the middle of the cyst wall, which corresponds to the peak s-WGA fluorescence intensity. Download FIG S2, TIF file, 1.9 MB. Copyright © 2020 Guevara et al. 2020 Guevara et al. This content is distributed under the terms of the Creative Commons Attribution 4.0 International license.

### The localization of s-WGA in mature cysts is not dependent on GRA2 expression.

**FIG 4:**
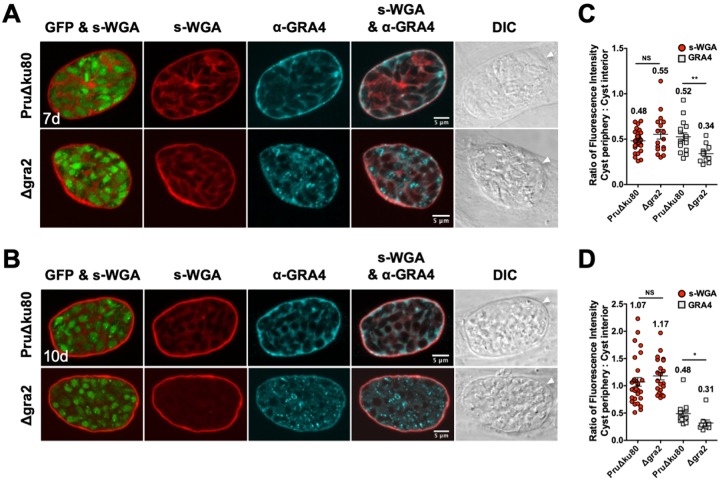
s-WGA colocalizes with GRA4 at the cyst periphery, and s-WGA accumulation at the cyst periphery is GRA2 independent in mature cysts. PruΔ*ku80*- and Δ*gra2*-infected HFF cells were treated under bradyzoite-inducing conditions to differentiate *in vitro* cysts for 7 days or 10 days. Cysts were located using DIC microscopy and imaged by confocal microscopy. (A and B) The presence of bradyzoites inside cysts was verified by locating parasite nuclei with DAPI staining (not shown) and verifying that each parasite nucleus was surrounded by the expression of cytosolic GFP (GFP^+^ bradyzoites). Cysts were stained with biotinylated s-WGA and anti-GRA4 antibody. Panels show GFP and s-WGA, s-WGA, anti-GRA4, s-WGA and anti-GRA4, and DIC (cyst walls are indicated by white arrowheads). s-WGA was colocalized with GRA4 in 100% of cysts evaluated at day 7 (*n *= 16) (A) and day 10 (*n *= 14) (B). (C and D) Fluorescence intensities of s-WGA and GRA4 were measured at the cyst periphery and within the cyst (cyst interior) in PruΔ*ku80* and Δ*gra2* cysts. Data are plotted as the ratio of the mean fluorescence intensity at the cyst periphery to that within the cyst interior ± SEM. The numbers of cysts analyzed for the parental PruΔ*ku80* and Δ*gra2* strains for s-WGA and GRA4 are as follows: s-WGA (*n* = 31, *n* = 17 cysts) and GRA4 (*n* = 16, *n* = 10 cysts) at day 7 (C), and s-WGA (*n* = 30, *n* = 21 cysts) and GRA4 (*n* = 14, *n* = 9 cysts) at day 10 (D). The numerical ratio for the mean fluorescence intensity is labeled for s-WGA and GRA4. *P* values were calculated using Student’s *t* test (*, *P < *0.05; **, *P < *0.01; NS, not significant).

**FIG 5:**
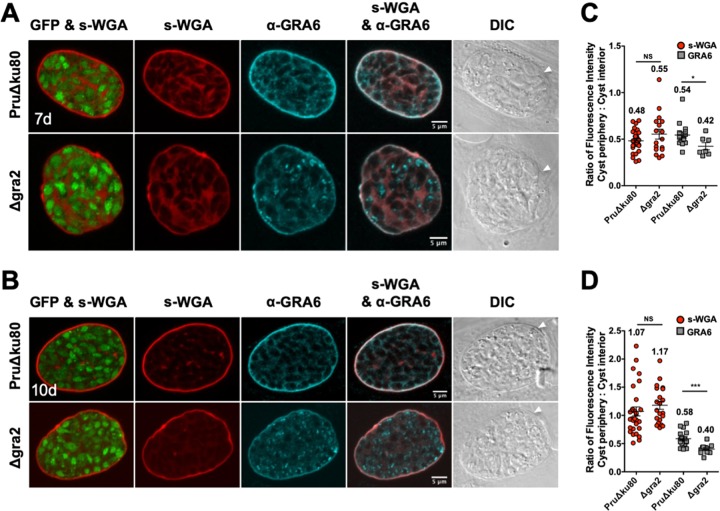
s-WGA colocalizes with GRA6 at the cyst periphery, and s-WGA accumulation at the cyst periphery is GRA2 independent in mature cysts. PruΔ*ku80*- and Δ*gra2*-infected HFF cells were treated under bradyzoite-inducing conditions to differentiate *in vitro* cysts for 7 days or 10 days. Cysts were located using DIC microscopy and imaged by confocal microscopy. (A and B) The presence of bradyzoites inside cysts was verified by locating parasite nuclei with DAPI staining (not shown) and verifying that each parasite nucleus was surrounded by the expression of cytosolic GFP (GFP^+^ bradyzoites). Cysts were stained with biotinylated s-WGA and anti-GRA6 antibody. Panels show GFP and s-WGA, s-WGA, anti-GRA6, s-WGA and anti-GRA6, and DIC (cyst walls are indicated by white arrowheads). s-WGA was colocalized with GRA6 in 100% of cysts evaluated at day 7 (*n *= 15) (A) and day 10 (*n *= 16) (B). (C and D) Fluorescence intensities of s-WGA and GRA6 were measured at the cyst periphery and within the cyst (cyst interior) in PruΔ*ku80* and Δ*gra2* cysts. Data are plotted as the ratio of the mean fluorescence intensity at the cyst periphery to that within the cyst interior ± SEM. The numbers of cysts analyzed for the parental PruΔ*ku80* and Δ*gra2* strains for s-WGA and GRA6 are as follows: s-WGA (*n* = 31, *n* = 17 cysts) and GRA6 (*n* = 15, *n* = 7 cysts) at day 7 (C), and s-WGA (*n* = 30, *n* = 21 cysts) and GRA6 (*n* = 16, *n* = 12 cysts) at day 10 (D). The numerical ratio for the mean fluorescence intensity is labeled for s-WGA and GRA6. *P* values were calculated using Student’s *t* test (*, *P < *0.05; ***, *P < *0.005; NS, not significant).

Mature cysts were differentiated for 7 days or 10 days, and the colocalization of s-WGA with GRA4 (Fig. 4) or GRA6 (Fig. 5) was measured. GFP^+^ bradyzoites were visible in s-WGA-stained cysts. In parental PruΔ*ku80* cysts, GRA4 (Fig. 4A and B) and GRA6 (Fig. 5A and B) colocalized with s-WGA at the cyst wall as well as in a continuous matrix pattern in the cyst matrix. This colocalization pattern was markedly reduced in Δ*gra2* cysts. The cyst periphery/cyst interior fluorescence intensity ratios of s-WGA (0.48) (Fig. 4C and Fig. 5C), GRA4 (0.52) (Fig. 4C), and GRA6 (0.54) (Fig. 5C) were similar in parental PruΔ*ku80* 7-day-old cysts, indicating cyst wall and cyst matrix localizations (15). The fluorescence intensity ratios of GRA4 (0.48) (Fig. 4D) and GRA6 (0.58) (Fig. 5D) in 10-day-old cysts remained similar to those in 7-day-old cysts, whereas the s-WGA ratio (Fig. 4D and Fig. 5D) was increased to 1.07, indicating the accumulation of s-WGA binding molecules in the cyst wall of 10-day-old cysts. In contrast to GRA4 and GRA6, the cyst periphery/cyst interior fluorescence intensity ratio of s-WGA was not affected in Δ*gra2* cysts. The fluorescence intensity peak of GRA4 (Fig. S3A and B) was shifted toward the cyst matrix in comparison to s-WGA. In contrast, the GRA6 (Fig. S3C and D) peak overlapped that of s-WGA, suggesting that s-WGA, like GRA6, was present in all layers of the cyst wall, whereas GRA4 primarily occupies the inner layers of the cyst wall.

10.1128/mSphere.00031-20.3 FIG S3 s-WGA accumulation at the cyst wall and distribution in the cyst matrix are GRA2 independent in mature cysts. Fluorescence intensity profiles of representative cysts, shown in Fig. 4A and B for GRA4 and in Fig. 5A and B for GRA6, were generated to quantify the location of s-WGA relative to GRA4 (A and B) or GRA6 (C and D) at the cyst wall at day 7 and day 10 in parental PruΔ*ku80* and Δ*gra2* strains. Dotted black lines define the cyst wall region. The dotted red line indicates the middle of the cyst wall, which corresponds to the peak s-WGA fluorescence intensity. Download FIG S3, TIF file, 1.3 MB. Copyright © 2020 Guevara et al. 2020 Guevara et al. This content is distributed under the terms of the Creative Commons Attribution 4.0 International license.

### s-WGA colocalizes with CST1 in the cyst wall.

**FIG 6:**
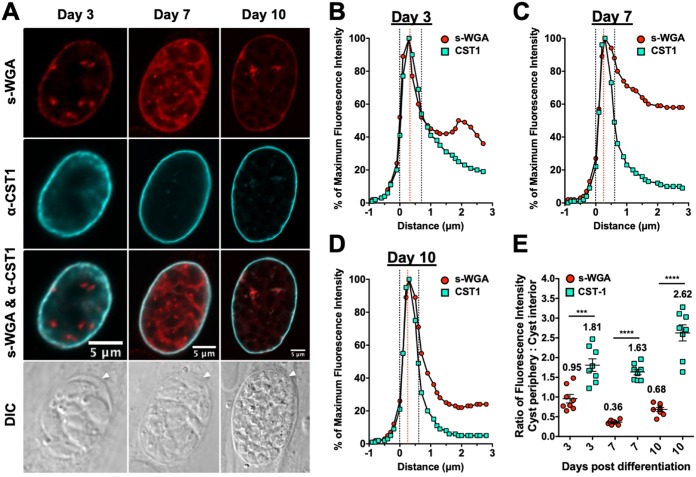
s-WGA colocalizes with CST1 at the cyst wall. PruΔ*ku80*-infected HFF cells were treated under bradyzoite-inducing conditions to differentiate *in vitro* cysts for 3 days, 7 days, or 10 days. Cysts were located using DIC microscopy and imaged by confocal microscopy. (A) The presence of bradyzoites inside cysts was verified by locating parasite nuclei with DAPI staining and verifying that each parasite nucleus was surrounded by the expression of cytosolic GFP (GFP^+^ bradyzoites) (not shown). Cysts were stained with biotinylated s-WGA and anti-CST1 antibody. Panels show GFP and DAPI, s-WGA, anti-CST1, s-WGA and anti-CST1, and DIC (cyst walls are indicated by white arrowheads). s-WGA was colocalized with CST1 at 3 days (*n *= 8), 7 days (*n *= 8), or 10 days (*n *= 8) in 100% of cysts evaluated. (B to D) Fluorescence intensity profiles of representative cysts shown in panel A were generated to quantify the location of s-WGA relative to the cyst wall as shown by CST1 at day 3 (B), day 7 (C), and day 10 (D). Dotted black lines define the cyst wall region. The dotted red line indicates the middle of the cyst wall. (E) Fluorescence intensities of s-WGA and CST1 were measured at the cyst periphery and within the cyst (cyst interior) at day 3, day 7, and day 10. Data are plotted as the ratio of the mean fluorescence intensity at the cyst periphery to that within the cyst interior ± SEM (*n *= 8 cysts). The numerical ratio for the mean fluorescence intensity is labeled for s-WGA and CST1 for each day shown. *P* values were calculated using Student’s *t* test (***, *P < *0.005; ****, *P < *0.0001).

We detected the colocalization of s-WGA binding molecules in the cyst wall with GRA4 and GRA6 in immature and mature cysts. To investigate the localization of s-WGA in relation to the major cyst wall protein CST1, we differentiated cysts for 3, 7, or 10 days and determined the localizations of s-WGA and CST1. Nuclei stained with 4′,6-diamidino-2-phenylindole (DAPI) were visible inside GFP^+^ bradyzoites (Fig. 6). As expected, CST1 localized to the cyst wall, while s-WGA localized to the cyst periphery/wall as well as to the cyst matrix in immature 3-day-old cysts and in mature 7- and 10-day-old cysts. Colocalization of CST1 and s-WGA was detected at the cyst periphery (Fig. 6A). To confirm that CST1 and s-WGA were colocalized in the cyst wall, we measured the cyst fluorescence intensity profiles for CST1 and s-WGA. CST1 was present in all layers of the cyst wall and colocalized with s-WGA in 3-day-old (Fig. 6B), 7-day-old (Fig. 6C), and 10-day-old (Fig. 6D) cysts. We quantitatively measured CST1 and s-WGA fluorescence intensities at the cyst periphery compared to those at the cyst interior. While CST1 was preferentially localized to the cyst wall (cyst periphery/cyst interior ratio of >1.0), the *N-*acetylglucosamine-modified molecules that bind s-WGA were present in the cyst wall and in the cyst matrix (cyst periphery/cyst interior ratio of <1.0) (Fig. 6E).

## DISCUSSION

Protozoan parasites possess survival strategies to maintain viability inside and outside the host(s) by forming cyst structures (38, 39). Neurons are the primary target cells for *Toxoplasma* cyst formation within distal neuronal processes (40, 41). Bradyzoite differentiation is triggered by changes in nutrition or cellular conditions (42–44), which drives the transcriptional program that leads to the production of bradyzoite-stage proteins (45, 46) and the formation of the cyst wall and cyst structure (32). The cyst wall is a protective structure that safely encloses bradyzoites until their transmission to a new host after oral ingestion of cysts (14). Glycosylated proteins are a major component of the cyst wall (19), which is an obstacle for cyst clearance and elimination by the host (47).

Tachyzoite-to-bradyzoite differentiation is linked with major changes in carbohydrate and polysaccharide metabolism (38, 48, 49). The *Toxoplasma* cyst wall is recognized by a variety of lectins, including concanavalin A (α-d-mannose or α-d-glucose), wheat germ agglutinin (WGA) (*N*-acetylglucosamine or sialic acid), succinylated WGA (s-WGA) (*N*-acetylglucosamine), soybean agglutinin (α-helix- or β-sheet-linked *N-*acetyl-d-galactosamine or galactose), and Dolichos biflorus, Vicia villosa, Artocarpus integrifolia, and Helix pomatia agglutinins (*N*-acetylgalactosamine) (22, 24, 26). The most widely used cyst wall stain is Dolichos biflorus agglutinin (DBA), which intensely stains the cyst wall of *in vitro* (31) and *in vivo* (15, 19, 23, 50–52) cysts. DBA selectively recognizes *O*-linked *N*-acetylgalactosamine glycosylation of the CST1 mucin domain (19). *O*-linked glycosylation of CST1 is essential for establishing cyst wall thickness and cyst stability (19, 23). *N*-Acetylgalactosamine-modified CST1 has been hypothesized to be a key cyst wall scaffolding molecule that interacts with other cyst wall proteins to provide a walled structure that supports cyst stability (13, 53). Our results show that the cyst wall-glycosylated molecule(s) that binds s-WGA colocalized with CST1 in the cyst wall. Together, *N*-acetylgalactosamine-modified CST1- and *N-*acetylglucosamine-modified s-WGA-interacting molecules may coordinate the scaffolding and building of the cyst wall.

Bright s-WGA-stained puncta were visible at the cyst periphery in 6-h-differentiated cysts, suggesting that *N*-acetylglucosamine-modified molecules preferentially accumulated at the cyst periphery/wall early after differentiation. This phenotype mirrors the recent phenotypes that we reported for DBA-stained cyst wall glycoprotein(s) (CST1) and the cyst wall components GRA1, GRA2, GRA4, GRA5, GRA6, GRA7, GRA9, and GRA12 (12, 15). The bright puncta present at the cyst periphery 6 h after differentiation may mark the presence of vesicles secreted by bradyzoites that carry cyst wall cargo for wall building to the cyst periphery. The actin cytoskeleton and Rab11 traffic vesicles to the cyst walls of *Giardia* and *Entamoeba*, supporting a model whereby cyst wall proteins are transported through the cytoskeleton (54, 55). In addition, the *Giardia* Rho GTPase Rac regulates endomembrane organization and cyst wall protein trafficking in *Giardia* (56). How *Toxoplasma* cyst wall cargo for cyst wall building is delivered to the cyst periphery is currently unknown. Future studies with electron and high-resolution microscopy are needed to identify the mechanisms that could traffic cyst wall cargo to the cyst wall.

After differentiation is triggered, the initial tachyzoite-stage PVM develops into the limiting cyst membrane (12). The limiting cyst membrane is ruffled (13, 16, 19). Cyst wall proteins accumulate at the cyst periphery during cyst wall development and localize in the cyst wall beneath the limiting cyst membrane (12, 15). The cyst wall structure has a densely compacted outer layer and a less densely compacted inner layer that faces the cyst matrix and is filled with vesicles and membrane tubules (13). The fluorescence intensity peak of s-WGA overlapped that of CST1, the major cyst wall glycoprotein that is localized throughout the cyst wall (15, 19, 23). Thus, the *N*-acetylglucosamine-modified molecules that bind to s-WGA are present throughout the cyst wall. Furthermore, s-WGA was preferentially localized in the cyst wall early after differentiation at 6 h, 1 day, and 2 days of cyst development. These results support the hypothesis that *N*-acetylglucosamine-modified molecules that bind to s-WGA and colocalize with CST1 play a role in the development and structure of the cyst wall. Genetic deletion of CST1 results in a leaky-cyst phenotype marked by the escape of cyst matrix proteins from the cyst (53), which supports the previously proposed model that *O*-linked *N*-acetylgalactosamine glycosylation regulates the permeability of the cyst wall. It is tempting to speculate that *N*-acetylgalactosamine-modified CST1- and *N-*acetylglucosamine-modified s-WGA binding molecules together establish permeability conduits in the cyst wall that allow the permeation of small essential host molecules such as glucose (57) and nucleotide sugars (27) and, perhaps, vesicular traffic. Additional studies are necessary to identify the structures formed by colocalized CST1- and s-WGA-binding molecules in the cyst wall.

The genetic deletion of GRA2 disrupted the organization of the cyst matrix, altered the staining patterns of GRA4 and GRA6 in the cyst matrix, and delayed the accumulation of GRA4, GRA6, and DBA-stained CST1 in the cyst wall (15). In the tachyzoite-stage PV, GRA2 tubulates vesicles into highly curved membranes, the intravacuolar network (IVN), that connect tachyzoites to one another and to the PVM (21, 58, 59). Similar highly curved membranes, the intracyst network (ICN), are also visible in the cyst matrix and in the cyst wall and connect the bradyzoites to one another and to the cyst wall (13, 14). While s-WGA preferentially stained the cyst wall early after differentiation prior to the formation of the cyst matrix, s-WGA prominently stained both the cyst wall and the cyst matrix of 3-, 7-, and 10-day-old cysts. s-WGA colocalized with GRA4 and GRA6 in the cyst matrix and with GRA4, GRA6, and CST1 in the cyst wall. In contrast to GRA4, GRA6, and DBA-stained CST1 (15), the deletion of GRA2 did not affect s-WGA localization in the cyst matrix or in the cyst wall. Thus, while s-WGA colocalized with GRA4, GRA6, and CST1, which traffic to the cyst wall via a GRA2-dependent pathway (15), the *N*-acetylglucosamine-modified molecule(s) that binds s-WGA traffics to and accumulates at the cyst wall through GRA2-independent mechanisms.

Glycan modifications in the tachyzoite stage have been previously reported to regulate invasion, O_2_ sensing, nutrient storage, and *in vitro* growth (60). Skp1 is required for cytoplasmic glycosylation and optimal oxygen-dependent growth (61, 62). Spy (TGME49_273500) (63) is an enzyme similar to *O*-linked *N*-acetylglucosamine transferase (OGT) that mediates *O*-linked β-*N*-acetylglucosaminylation. *O*-linked β-*N*-acetylglucosaminylation is a posttranslational modification of cytosolic, nuclear, and mitochondrial proteins by a single residue of *N*-acetylglucosamine which is transferred from UDP-*N*-acetylglucosamine and is hypothesized to be involved in the localization of nuclear proteins (64, 65). *O*-linked β-*N*-acetylglucosaminylation is reversible by *O*-linked β-*N*-acetylglucosaminyl hydrolase (OGA), which removes the *N-*acetylglucosamine residue (66). *O*-linked β-*N*-acetylglucosaminylation levels are dependent on nutritional status (67), and since starvation triggers the tachyzoite-to-bradyzoite transition (42), it is postulated that *O*-linked *N*-acetylglucosamine glycosylation levels would be increased in bradyzoites. In the tachyzoite stage, a variety of *Toxoplasma* proteins are modified with *N*-acetylglucosamine (65), and the tachyzoite proteome of *O*-linked β-*N*-acetylglucosaminylation was recently determined (68). s-WGA binds to many tachyzoite-stage-expressed proteins that are glycosylated via *O*-linked *N*-acetylglucosamine residues (68), and our results suggest that *O*-linked β-*N*-acetylglucosaminylation may also occur in the bradyzoite/cyst stage.

The chitin-binding s-WGA lectin specifically recognizes *N*-acetylglucosamine-modified structures and does not bind to sialic acid residues like the WGA lectin (29). Previously, cysts were thought to possess chitin in the cyst wall based on evidence showing that (i) chitinase-treated cysts failed to bind s-WGA (24) and (ii) the cyst wall of chitinase-treated cysts was degraded, and bradyzoites were released (24, 30). These results demonstrated that s-WGA binding to the cyst wall is chitinase sensitive and support the hypothesis that the cyst wall-localized *N*-acetylglucosamine-modified molecule(s) recognized by s-WGA and chitinase is essential for the stability of the cyst wall and cysts. Thus, the molecules recognized by s-WGA and chitinase and the biological functions of these cyst wall-localized molecules are likely to be potential targets for cyst disruption and cyst elimination.

The only confirmed cyst wall molecule that is recognized and bound by s-WGA is an uncharacterized ∼48-kDa cyst wall glycoprotein (23). Chitinases are glycosyl hydrolases that cleave the β-1,4 linkage of *N*-acetylglucosamine that is present in chitin and chitin-like oligosaccharide structures. The *Toxoplasma* cyst wall as well as the oocyst wall most likely do not contain chitin in view of the fact that *Toxoplasma* as well as its mammalian hosts do not express a chitin synthase (69, 70). Chitinase will not recognize *O*-linked *N*-acetylglucosamine residues since the requisite β-1,4 *N*-acetylglucosamine linkages required for chitinase activity are absent in *O*-linked glycoprotein modifications. Intriguingly, similar to the behavior of *Toxoplasma* cysts, chitinase treatment eliminated s-WGA/WGA binding of *Giardia* cysts (71). *Giardia* cysts also do not contain chitin, and the recognition of the *Giardia* cyst wall by s-WGA and WGA was previously attributed to the presence of short *N*-glycans in the cyst wall (70). *Toxoplasma* also has the capability of *N*-glycan modification of glycoproteins (72), although *N*-glycan modification in the cyst stage has not been previously investigated. It is plausible that the ∼48-kDa *Toxoplasma* cyst wall glycoprotein that selectively binds s-WGA (23) is posttranslationally modified by *N*-linked glycosylation that is recognized by s-WGA in a chitinase-sensitive manner. This hypothesis remains to be tested, as is whether the putative *N*-glycans that bind s-WGA and are recognized by chitinase are short *N*-glycans or bisected hybrid-type *N*-glycans with the chitobiose core of the oligosaccharide GlcNAcβ1-4Manβ1-4GlcNAcβ1-4GlcNAc, which is known to be avidly bound by s-WGA and WGA (73, 74).

## MATERIALS AND METHODS

### Culture conditions and strains.

Type II Prugniaud (Pru)-background Toxoplasma gondii parasites were maintained *in vitro* by serial passage of tachyzoites in human foreskin fibroblast (HFF) monolayers (ATCC SCRS-1041.1) cultured in Eagle’s modified essential medium (EMEM) (Lonza) containing 1% fetal bovine serum (FBS) (Life Technologies), 2 mM glutamine, 100 U/ml penicillin, and 100 μg/ml streptomycin at 36°C in 95% air and 5% CO_2_. HFF cells were maintained in EMEM containing 10% FBS (HyClone), 2 mM glutamine, 100 U/ml penicillin, and 100 μg/ml streptomycin at 37°C in 95% air and 5% CO_2_. The parental Pru strain PruΔ*ku80* was previously made transgenic for green fluorescent protein (GFP) under the control of the LDH2 bradyzoite-stage-specific promoter (75). The Δ*gra2* strain was previously developed using the PruΔ*ku80* knockout strain of the type II Pru strain (17, 76).

### *In vitro* cyst differentiation assay.

Tachyzoites were differentiated *in vitro* into bradyzoites within cysts essentially as previously and elegantly described by Tobin and colleagues (31). Differentiation medium contained RPMI medium without bicarbonate supplemented with 2.05 mM l-glutamine (HyClone), 20 mM HEPES-free acid (IBI Scientific), 1% XL-glutamine (a long-lasting stable form of glutamine; VWR), 1% FBS, and 1% penicillin-streptomycin. The pH of differentiation medium was adjusted to 8.1 with sodium hydroxide and the medium was filter sterilized. HFF cells were cultured on circular micro cover glass until confluent (Electron Microscopy Sciences), and confluent monolayers were infected with type II Pru parasites at a multiplicity of infection (MOI) of ∼0.5. Infected HFF cells were washed once 3 h after infection in Dulbecco’s phosphate-buffered saline (DPBS) supplemented with Ca^2+^ and Mg^2+^ and incubated in differentiation medium for 6 h, 1 day, 2 days, 3 days, 7 days, or 10 days at 37°C in ambient air. Medium was changed on day 3 and day 7.

### Cyst immunofluorescence assay and cyst location.

Infected cells were fixed in 4% paraformaldehyde for 10 min, and the excess was quenched with 0.1 M glycine. Infected cells were permeabilized and blocked in 3% FBS–0.2% Triton X-100 for 30 min, and this solution was used in all proceeding incubation steps. All samples were incubated with a 1:500 dilution of biotinylated, succinylated WGA (Vector Laboratories); a 1:25 dilution of primary mouse monoclonal anti-CST1 antibody (23); a 1:1,000 dilution of primary mouse monoclonal anti-GRA2 antibody (77); or a 1:1,000 dilution of primary rabbit anti-GRA4 (20) or anti-GRA6 (20) (antibodies were purchased from the Biotem Company, Apprieu, France, or kindly provided by L. D. Sibley, Washington University School of Medicine, St. Louis, MO, or L. M. Weiss, Albert Einstein College of Medicine, Bronx, NY). Preparations were washed three times with DPBS supplemented with Ca^2+^ and Mg^2+^ and incubated for 1 h at room temperature (RT) with a 1:1,000 dilution of secondary goat anti-rabbit IgG(H+L) (Thermo Fisher) and goat anti-mouse IgG(H+L) antibodies conjugated to Alexa Fluor 647 (Cell Signaling). All samples were incubated with a 1:200 dilution of DyLight 594 streptavidin (Vector Laboratories) for 1 h at RT. Samples were mounted in SlowFade gold antifade with 4′,6-diamidino-2-phenylindole (DAPI) (Life Technologies) and then imaged with a Nikon A1R SI confocal microscope (Nikon, Inc.) using an Apo total internal-reflection fluorescence (TIRF) 100× oil differential interference contrast (DIC) N20 objective. Cysts were randomly selected for analysis by locating cysts using DIC microscopy. Bradyzoite differentiation in cysts was confirmed by GFP^+^ bradyzoites. The focal plane (from a z-stack) selected for quantification was from the middle of the cyst, where the cyst size is maximal. Raw .nd2 files of cyst images were imported into Fiji for processing. Images were minimally processed for brightness (image → adjust → color balance) in Fiji (78). The colocalization of s-WGA with GRA4, GRA6, or CST1 was determined visually by analyzing each imaged cyst for spots of fluorescence overlap, and the percentage was calculated by the images that showed colocalization out of the total number of cysts imaged. The number of cysts for each strain analyzed in each experiment is reported in each figure legend.

### Cyst fluorescence intensity profiles.

Raw .nd2 image files were imported into Fiji to measure fluorescence intensity parallel to the cyst wall, as previously described (15). Images were cropped to isolate each cyst. A macro was written to generate a reliable mask of the cyst, slightly outside the cyst wall, using the s-WGA channel. The s-WGA channel was used to threshold the cyst, and holes were filled inside to obtain a continuous mask of the whole cyst. Successive layers were generated based on the original mask, growing or shrinking by dilate or erode morphological operations. Layers were generally 1 pixel thick. The fluorescence intensity of each region was measured for a selected fluorescence channel: s-WGA, CST1, GRA4, or GRA6. The macro generated layers within the cyst until the minimum area of the (shrinking) layer reached 1,000 square pixels. Layers were created by dilation to measure the fluorescence intensity outside the cyst, which provided the background fluorescence intensity. All data were imported into Excel to be further analyzed, as previously described (15). The calculated percent maximum fluorescence intensity and distance (micrometer) values were imported and graphed in Prism.

### Cyst wall definition and analysis.

The cyst wall region was identified and defined as previously described (15). The cyst wall outer region was identified by s-WGA, while the inner region was determined by GFP, which identifies the parasites within the cyst. The cyst wall region is defined by an outer and an inner boundary determined by the first point with <50% of the maximum fluorescence intensities of s-WGA and GFP, respectively. The cyst wall region is marked by dotted black lines, and the peak of s-WGA fluorescence is marked by a dotted red line. Next, we evaluated the location of CST1, GRA4, or GRA6 in comparison to the s-WGA-stained cyst wall using fluorescence intensities measured at the same time within the cyst. This cyst wall analysis was used to determine if two proteins were observed in the same layer.

### Cyst total fluorescence intensity quantification assay.

Raw .nd2 image files were imported into Fiji to measure the total fluorescence intensity at the cyst periphery and within the cyst interior, as previously described (15). The cyst periphery was determined to be the cyst wall plus two layers, which were added to include proteins near the cyst wall but not yet incorporated into the cyst wall. Fluorescence for s-WGA, CST1, GRA4, and GRA6 was measured in the Δ*ku80* and Δ*gra2* strains. To measure background fluorescence, a circle was drawn using the freehand selection tool, and fluorescence was measured outside the cyst on three different sides. All data were imported into Excel to be further analyzed, as previously described (15). All ratios were entered and graphed in Prism. A ratio of <1 means that there is a higher s-WGA, CST1, or GRA fluorescence intensity in the cyst interior than at the cyst periphery; a ratio of 1 represents s-WGA, CST1, or GRA fluorescence intensity at the cyst periphery equal to that in the cyst interior; and a ratio of >1 means that there is a higher s-WGA, CST1, or GRA fluorescence intensity at the cyst periphery than in the cyst interior. *P* values were calculated using Student’s *t* test.

### Statistical analysis.

Unpaired *t* tests were used to calculate *P* values. All calculations of averages ± standard errors of the means (SEM) and *P* values were performed using GraphPad Prism software version 5.0c.
