# Supplementary figures and images for: Succinylated Wheat Germ Agglutinin Colocalizes with the Toxoplasma gondii Cyst Wall Glycoprotein CST1
Source: mSphere. 2020 Mar 4;5(2):e00031-20. doi: 10.1128/mSphere.00031-20 (PMC7056803; doi:10.1128/mSphere.00031-20)

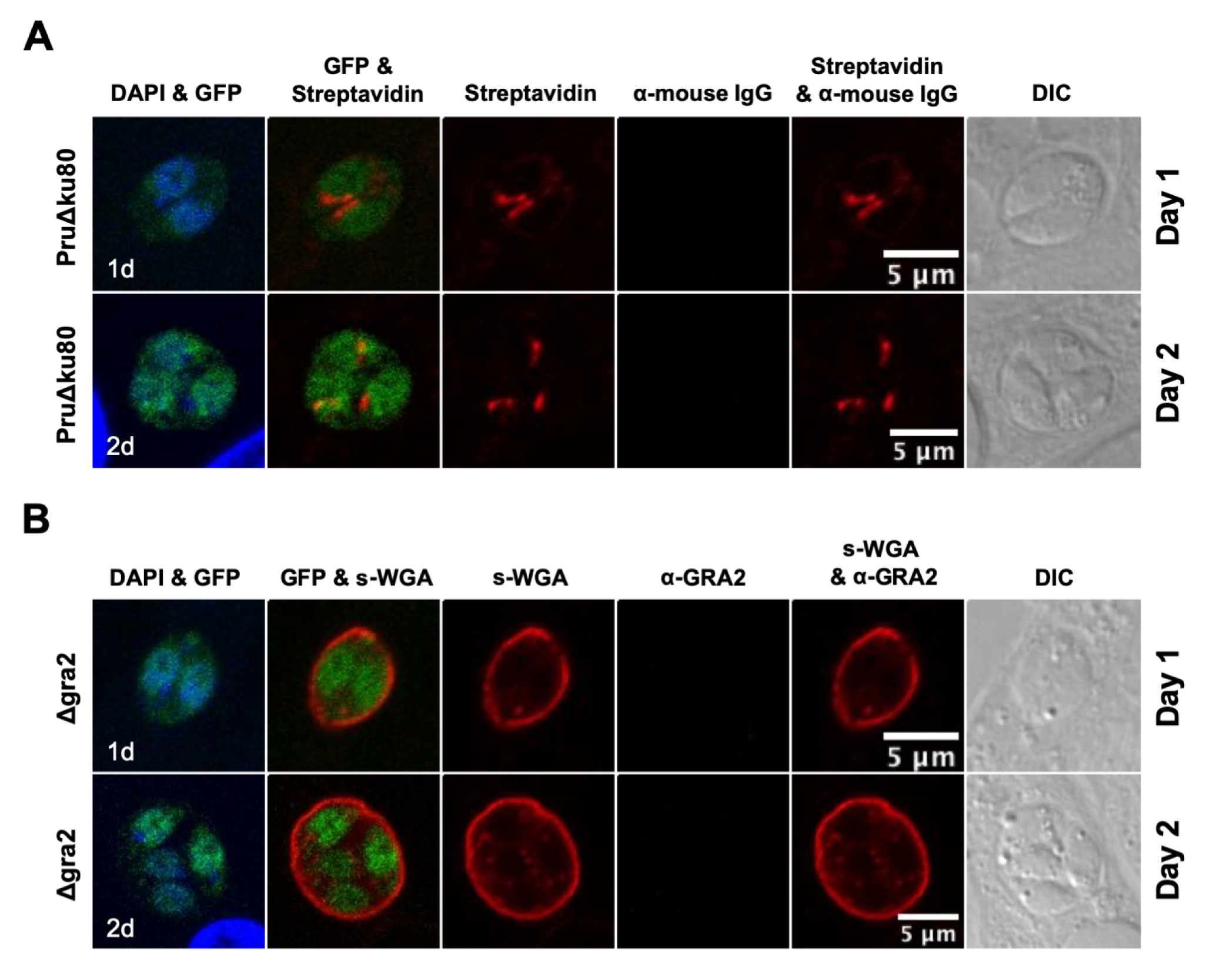

Supplement: FIG S1 [file mSphere.00031-20-sf001.tif]

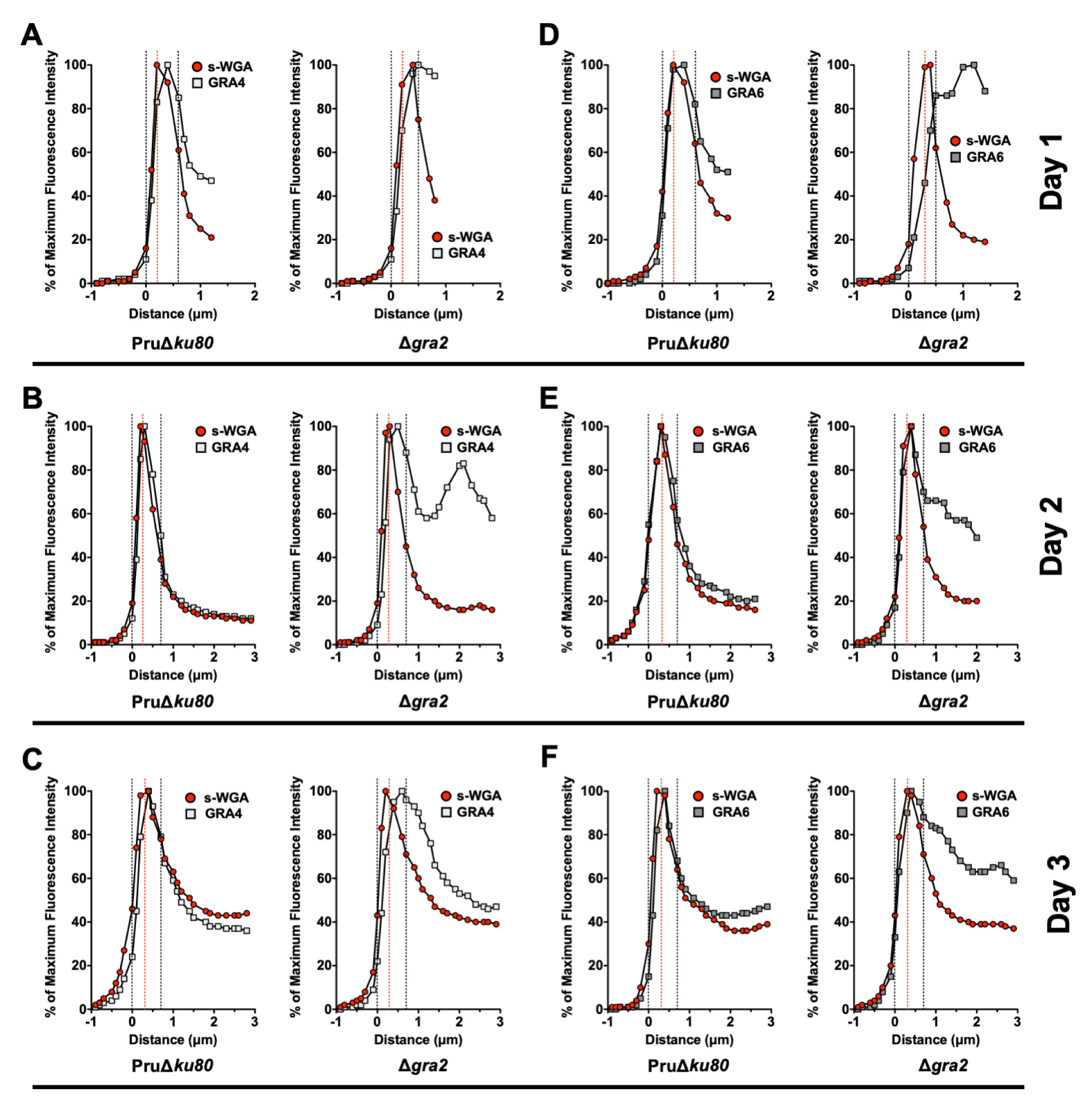

Supplement: FIG S2 [file mSphere.00031-20-sf002.tif]

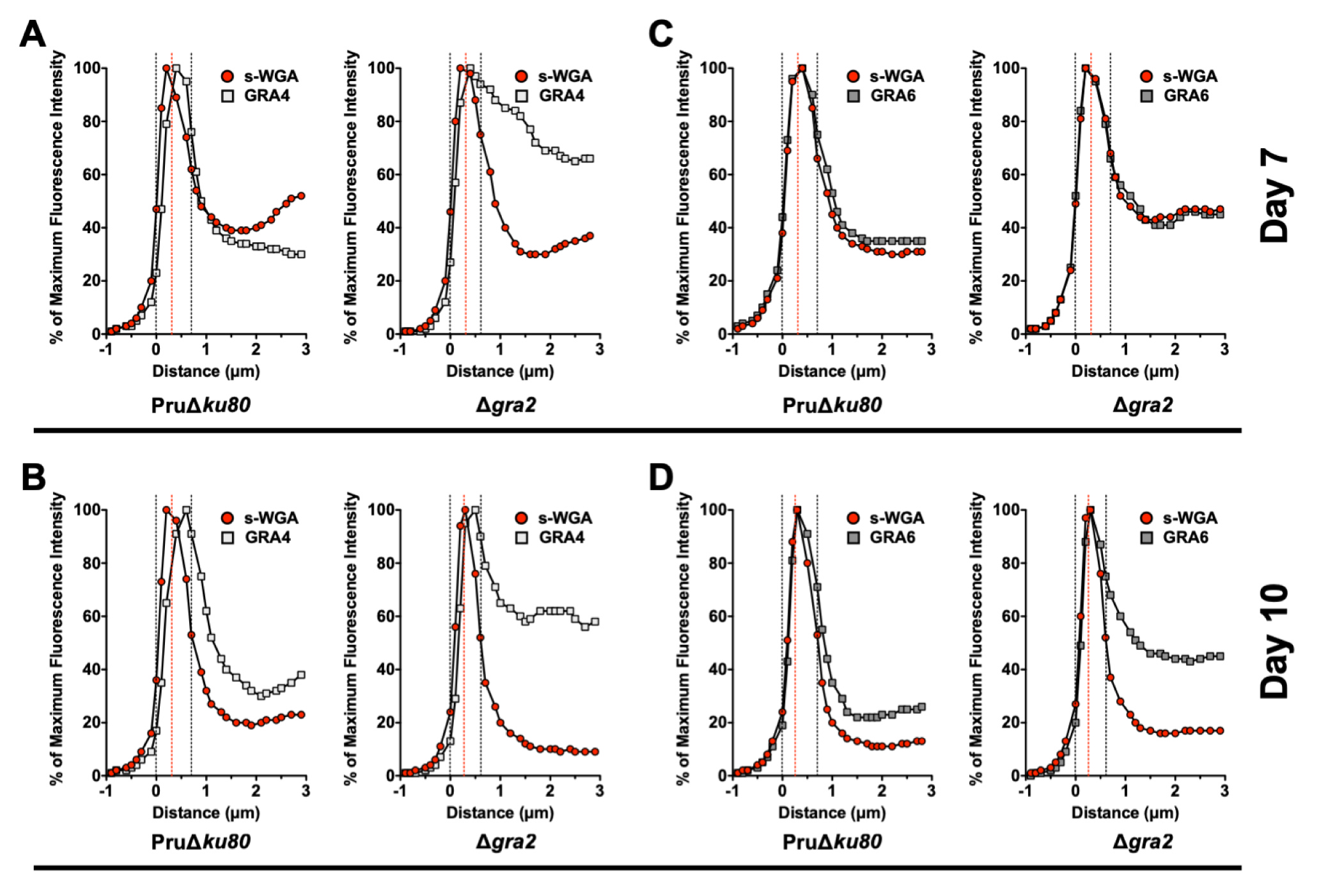

Supplement: FIG S3 [file mSphere.00031-20-sf003.tif]
